# Sterile post-traumatic immunosuppression

**DOI:** 10.1038/cti.2016.13

**Published:** 2016-04-29

**Authors:** Md Nahidul Islam, Benjamin A Bradley, Rhodri Ceredig

**Affiliations:** 1 Regenerative Medicine Institute, National Centre of Biomedical Engineering Science and School of Medicine, Nursing and Health Sciences, National University of Ireland, Galway, Galway, Ireland; 2 Orthopaedic Research Unit, Learning and Research Building, Southmead Hospital, University of Bristol, Bristol, UK

## Abstract

After major trauma, the human immune system initiates a series of inflammatory events at the injury site that is later followed by suppression of local inflammation favoring the repair and remodeling of the damaged tissues. This local immune response involves complex interactions between resident cells such as macrophages and dendritic cells, soluble mediators such as cytokines and chemokines, and recruited cells such as neutrophils, monocytes and mesenchymal stromal cells. If of sufficient magnitude, these initial immune responses nevertheless have systemic consequences resulting in a state called post-traumatic immunosuppression (PTI). However, controversy exists regarding the exact immunological changes occurring in systemic compartments triggered by these local immune responses. PTI is one of the leading causes of post-surgical mortality and makes patients vulnerable to hospital-acquired infections, multiple organ failure and many other complications. In addition, hemorrhage, blood transfusion, immunesenescence and immunosuppressant drugs aggravate PTI. PTI has been intensively studied, but published results are frequently cloudy. The purpose of this review is to focus on the contributions made by different responsive modalities to immunosuppression following sterile trauma and to try to integrate these into an overall scheme of PTI.

## Definitions

***Immune status*** is the level of appropriately targeted resistance to internal, opportunistic and external pathogenic microorganisms, where resistance is attributable to multiple innate or acquired mechanisms located throughout the organism. There is no single measure of immune status, but rather multiple biomarkers that make up a profile describing broad aspects of innate and adaptive immunity.

***Post-traumatic*** ***immunosuppression*** (PTI), for the purpose of this review, is the condition of suppressed immune status that follows sterile trauma. It ranges from mild to severe, where mild is exemplified by strenuous exercise and severe by immunosuppression induced by multiple extensive trauma or major open surgery.

***Sterile trauma*** refers to tissue damage devoid of primary wound infection, and is best exemplified by elective open surgery, for example, in knee joint arthroplasty. Inflammation following sterile trauma without any exposure to microbial pathogens is termed ‘sterile inflammation'.^1^ Immunosuppression followed by this initial sterile inflammation termed as ‘sterile immunosuppression'.

***Non-sterile trauma*** refers to tissue damage due to sepsis or any injury/surgery in the presence of microbial infection. Inflammation following any trauma with exposure to microbial pathogens is termed ‘non-sterile inflammation'. Immunosuppression followed by initial non-sterile inflammation can be termed as non-sterile immunosuppression.

***Inflammation*** has different definitions

*Clinical concept* – John Hunter's outstanding surgical and experimental observations on ‘inflammation' suggested four clinical signs, namely, redness, heat, swelling and pain.^2^ However, Hunter's definition of ‘Inflammation' was not based on understanding the immunological responses.

*Immunological concept* – It is only relatively recently that inflammation has become an important term in immunology. The Pro-/Anti-inflammatory paradigm is the basic concept of inflammation reflected in a balance between the two opposing cytokine networks that activate or suppress immunity.

An evolutionarily sophisticated and balanced immune system exists in our body whose equilibrium can be altered by different physical, environmental or psychological stresses. Trauma, including major surgery and accidental injury, leads to PTI that increases a patient's vulnerability to hospital-acquired infections. However, the underlying mechanisms of PTI are poorly defined and as yet, there are no universally accepted treatments.

In 1856 Florence Nightingale drew attention to the ‘utter insignificance' of risk of dying from battle wounds acquired during the Crimean War compared with the risk of dying from subsequent zymotic (infectious) diseases acquired within the Scutari Hospital.^3^ Nightingale's continuous improvements in hospital hygiene gradually reduced deaths from infectious diseases in patients with combat injuries. However, more and more new infections are still threatening the battle wound patients.^4, 5^ An important, but poorly highlighted, question remains, namely, ‘why do wounded patients acquire systemic infections even in a hygienic environment?' Research over the past two decades suggests that an ‘imbalanced immunity in patients following trauma' is the most important factor in increasing a patient's vulnerability to acquire infections. Therefore, although sanitation has been improved in the hospitals, an equivalent phenomenon of post-traumatic deaths from systemic infections persists to this day. Recent *in vivo* studies confirmed the trauma-associated translocation of endogenous bacteria from the gut following sterile head injury,^6, 7^ indicating another source of PTI.

One attempt to conceptualize the phenomenon of PTI was the so-called SIRS–CARS paradigm, where trauma is described as a ‘systemic inflammatory response syndrome', followed by a ‘compensatory anti-inflammatory response syndrome'. In 1991, Dr Roger Bone introduced the term ‘systemic inflammatory response syndrome (SIRS)' to describe physiological changes common to all cases of burn, trauma or sepsis. This paradigm included four physiological changes, namely, increased body temperature, elevated heart rate, tachypnea or hyperventilation, and leukocytosis or lekocytopenia.^8^ Despite its obvious limitations, until now SIRS has been the dominant paradigm adopted by clinicians worldwide. For example, body temperature can increase following sepsis and sterile trauma, but also after myocardial infarction, pulmonary embolism and strenuous exercise. Elevated heart rate and tachypnea can occur following sepsis and sterile trauma, but other physiological complications such as cardiac and respiratory failures, hypovolemic shock, and erythropenia can also affect these parameters. On the other hand, alteration in white blood cell count can also happen in different disease conditions such as heart failure, pancreatitis and burns. In 1996, bone modified his SIRS paradigm by adding a sequel named ‘compensatory anti-inflammatory response syndrome (CARS)'. CARS was characterized by a decrease in antigen presentation, macrophage paralysis, decrease in T-cell proliferation, increase in T-cell and dendritic cell (DC) apoptosis, and shift in the T-cell subsets from Th1 to Th2 phenotype.^9^ However, in 2002, these parameters were further modified with many additional criteria included, such as increases in C-reactive protein, creatinine, bilirubin or lactate, or hyperglycemia in the absence of diabetes,^10^ but in general their addition has resulted in more confusion in the field. Injured tissues release soluble factors that act on the endocrine, lymphoid and haematopoietic organs. Moreover, the SIRS–CARS criteria as initially proposed were poorly associated with immunofunctional parameters and recent observations failed to fit with this original paradigm.^11, 12, 13, 14, 15, 16^ Therefore, the SIRS–CARS concept may not be the best way of describing PTI.

Micro- versus macro-surgery (minor- versus major-injury) also has an important role in post-traumatic immunology and a patient's clinical outcome. Minimally invasive surgeries reduce complications compared with conventional operations. Considering the advantages of minimally invasive surgical procedures such as the requirement of smaller incisions, less analgesics, less injury scores and reduced stay in hospital, this form of surgery has become common practice. The severity of trauma is reflected in the amount of cytokine production^17, 18^ with little cytokine release following minimally invasive surgeries, such as laparoscopic procedures. Following major surgeries, such as arthroplasty or other colorectal/vascular procedures, higher levels of cytokines are released.

Investigations after major trauma, where venous blood samples are monitored, assume that the results obtained reflect systemic immunity, although in reality, much of the immune response is confined to locally injured tissues. Following the initial local inflammatory events that tend to increase tissue damage, there is activation of an anti-inflammatory cascade that helps local wound healing. This anti-inflammatory cascade is therefore a very important physiological event to maintain immune-homeostasis following sterile trauma. Rock *et al.*^19^ emphasize current limitations in our knowledge about the immunological consequences of sterile inflammation particularly the role of dead cells and their released sterile irritant particles in interleukin (IL)-1-dependent inflammasome activation. Thus, their review provided a concise account of the effects of different released particles from dying and necrotic cells on inflammation. Although sterile PTI is a complex event, little information is available on changes in the levels of different immune- and non-immune-soluble mediators, and cell types in this process.

Both sterile- and non-sterile trauma can trigger partly similar physiological responses. This review is therefore purposely restricted to discuss only the immunological events following sterile trauma.

## Immunological changes following sterile trauma

### Changes at local site

#### Skin, the primary barrier

Human skin acts as both a physical and, by local production of antimicrobial peptides, a chemical barrier against foreign microbial infections. Keratinocytes and resident cells of the innate immune system reduce the epidermal microbial load by triggering the release of antimicrobial peptides.^20^ Injury to the skin results in immediate hemostasis, followed by infiltration of neutrophils and macrophages and simultaneous production of cytokines such as IL-6 and IL-8.^21^ IL-6 has a role in fibroblast proliferation, collagen deposition and angiogenesis. Interestingly, a strong correlation exists between local IL-6 concentrations and the speed of wound healing.^22^

#### Ischemia/reperfusion-related changes

Major orthopedic surgery is frequently carried out under tourniquet, creating tissue hypoxia that, following tourniquet release, gives rise to ischemia–reperfusion injury to tissues. A key component of a cell's response to hypoxia is upregulation of hypoxia-induced transcription factor (HIF) expression^23^ In hypoxic conditions, increasing HIF and Toll-like receptor (TLR) signaling synergistically activates the NFkB pathway, thereby triggering the recruitment of monocytes, phagocytosis and release of IL-18, tumor necrosis factor (TNF)-α and damage-associated molecular patterns (DAMPs).^24^ HIF also inhibits apoptosis of recruited neutrophils at the site of injury, thereby prolonging their lifespan.^25^ However, HIF upregulation in T cells stimulates IL-10 production, inducing a shift from inflammatory Th1 phenotype to anti-inflammatory Th2 phenotype.^25^ Hypoxia increases the adenosine levels that regulate innate immunity and controls inflammation.^26^ Following tourniquet release, reperfusion injury may also enhance the expression of inflammatory cytokines such as IL-17 facilitating further recruitment of leukocytes to the site of injury;^27^ however, DAMP-driven immunological responses may reduce inflammatory events to facilitate healing.

#### Local changes in soluble mediators

Reviewing the published literature, considerable variation has been reported in the protein levels of different soluble mediators at the surgical wound site, summarized in Table 1 and detailed in Supplementary Table S1. There is general agreement that increased levels of IL-1β, IL-4, IL-6, IL-8, IL-10, TNF-α, PGE_2_ and complement proteins–C3 and C5–were seen postoperatively.

### Changes in hypothalamic–pituitary–adrenal axis

The response to sterile trauma involves activation of the hypothalamus–pituitary–adrenal (HPA) axis and subsequent changes in hormonal levels. There are complex functional inter-relationships between HPE and the immune system. Secretion of inflammatory cytokines such as IL-1, IL-6 and TNF-α has impacts on the HPA axis.^28^ Following trauma, release of these mediators triggers the secretion of corticotrophin-releasing factor, a hypothalamic-releasing substance by the anterior pituitary gland, that immediately stimulates the release of adrenocorticotropic hormone (ACTH).^13^ ACTH rapidly stimulates the production of the glucocorticoid cortisol. The production of ACTH following trauma overrides cortisol-dependent ACTH downregulation, ultimately resulting in continuous increases in both ACTH and cortisol.^13^ Cortisol triggers the release of anti-inflammatory mediators such as IL-6, IL-1 receptor antagonist (IL-1RA) and soluble TNF receptors that have an important role in controlling inflammatory events, thereby leading to systemic immunosuppression. Post-traumatic stress disorder, leading to PTI, alters the homeostatic balance between the HPA axis and immune system due to imbalanced secretions of both non-immune and immune molecules that are involved in these physiological mechanisms.^29^ Although the HPA axis and immune system are hugely inter-connected, little is known about the balance between these two axes in the clinical settings of post-traumatic stress disorder-induced PTI. Thus, mild traumatic brain injury (mTBI), another form of sterile trauma, may also lead to immunosuppression.^30^

### Changes in immune cells

Increases in the number of neutrophils, monocytes, and mesenchymal stromal cells (MSCs) in blood reflect recruitment of these cells from extravascular compartments (spleen and bone marrow) after sterile injury.^31, 32, 33^ In contrast, depending on the degree of injury, reduction in the levels of red blood cells and lymphocytes are seen.^34^ The effects of trauma on individual immune cell types are briefly discussed below:

#### Neutrophils

During sterile trauma, neutrophils egress from the extravascular spaces into the circulation and are recruited to the site of injury. This neutrophil egress is facilitated by the CXC chemokine receptors CXCR-4 and CXCR-2.^35^ CXCR-4 ligands are involved in retaining neutrophils in the bone marrow, but CXCR-2 ligands inhibit CXCR-4-dependant retention thereby encouraging egress.^31, 35^ Granulocyte colony-stimulating factor also helps in the mobilization of neutrophils by altering the balance between ligation of CXCR4 and CXCR2.^35^ Ubiquitin, an endogenous CXCR4 agonist, is also released following trauma and burns, controlling the extent of neutrophil release from the bone marrow to the site of injury.^36, 37^

Neutrophils in the peripheral blood can also be rapidly recruited to the site of injury.^38, 39^ By their interaction with different DAMPs, these sentinel cells release different inflammatory cytokines and chemo-attractants that recruit additional neutrophils to the inflammatory site.^39, 40^ Following sterile trauma, a huge number of immature neutrophils mobilize from bone marrow into the circulation. However, recent investigation on immune response by the immature neutrophils (by checking the ability of phagocytosis and chemotaxis and measuring the expressions of CD14, CD16, TLR-2 and TLR4) in patients with sterile SIRS, showed that, although immature, these cells can still perform their crucial task as a first line of defense.^41^ Studies also described the limitations of these immature neutrophils in the circulation following surgery.^42, 43^

#### Monocytes and DCs

The mechanisms involved in the egress of monocytes from the bone marrow to the injury site are still poorly understood. In mouse, Tsou *et al.*^32^ illustrated the recruitment of Ly-6^+^ monocytes from the bone marrow into the circulation by a CCR-2-mediated pathway. Additional investigation suggested the spleen as another reservoir of monocyte recruited to damaged tissues.^44^ Importantly, emigration of monocytes from the bone marrow to the injured site is independent of neutrophil recruitment.^45^

Decreased expression of HLA-DR by circulating monocytes was also found in patients following trauma, major surgery and burns.^34, 46^ Antigen-presenting cells (APCs), such as DCs, interact with lymphocytes to trigger adaptive immunity. Kawasaki *et al.*^47^ reported decreased antigen presentation capacity by splenic DC in mice following trauma/hemorrhage. This was associated with decreased expression of MHC class II, IL-12 and IFN-γ. The total number of DCs showed a transient increase after surgery, but decreased at postoperative days 2–3.^48^ Recent studies showed decreased myeloid DC (MDC) but no change in plasmacytoid DC (PDC) 3–5 days after surgery.^49^ MDCs may possibly have been recruited to the surgery site. Circulating MDCs but not plasmacytoid DCs were shown by Maier *et al.*^50^ to undergo apoptosis. Several studies on DCs from circulating blood showed increased expressions of genes for chemotaxis, including CCL5, CXCL5 and CXCL4, anti-apoptosis (such as TIMP-1 and BCL2) and inflammation (such as NF-κB).^50, 51, 52, 53^ These results may indicate a role for DC in the recruitment of innate immune cells at the injury site.

Myeloid-derived suppressor cells are a mixed population of myeloid cells capable of suppressing T-cell responses. The number of CD14^+^ APCs was found to be significantly elevated for at least 24 h following surgery with relatively greater increases in CD16^+^, CD80^+^ and CD86^+^ APCs.^54^ More importantly, there was a massive increase in the number of CD14^+^HLA-DR^−^ MDSCs,^55^ indicating their involvement in immunosuppression following trauma.^56^ However, further phenotypic and functional studies are needed to confirm the subtype of these CD14^+^ APCs and their role in trauma-induced immunological responses.

#### Mesenchymal stromal cell

Recent investigation show increased numbers of circulatory MSCs in the peripheral blood of burns and trauma patients.^33, 57^ There appears to be a direct correlation between the proliferation rate of cultured bone marrow MSCs from patients with multiple traumas and trauma severity.^33^ How MSC egress from bone marrow and are recruited to the injury site remains unclear.

#### Lymphocytes

A significant decrease in total CD3^+^ T lymphocytes following trauma is associated with a shift from Th1 to Th2 phenotype mediated by regulatory T cells.^46, 58^ Albertsmeier *et al.*^54^ showed a decrease in CD3^+^, CD4^+^ and CD28^+^ T-cell counts immediately after surgery with no changes in CD8+ T cells; CD4^+^CD25^+^CD127^−^ regulatory T cells involved in shifting the T-helper compartment from Th1 to Th2 phenotype were increased. They hypothesized that suppression of T cells following trauma is associated with increased regulatory T cells and myeloid-derived suppressor cells.^54^ *In vitro* stimulation studies also showed reduced secretion of IFN-γ, IL-2 and TNF-α by postoperative T cells.^46^ On the other hand, Munoz *et al.*^59^ showed no postoperative changes in CD19^+^ B-lymphocyte counts from the first postoperative day until seventh. Lymphopenia and also the deactivation of cells in stroke patients are indicative of immune-suppression following sterile trauma.^60^

#### Natural killer cells

The functional activity of natural killer (NK) cells as a first-line responder of innate immunity is decreased following traumatic injury.^61, 62^ The number of CD16^+^CD56^+^ NK cells is decreased 1 day after knee joint replacement surgery. This decrease persisted until 1 week after surgery.^59^ Gharehbaghian *et al.*^61^ also showed significant suppression of NKp frequencies 5 days following joint replacement surgery; however, the exact CD phenotype of such NKp was not determined.

#### Other cells

Postoperative eosinophil counts initially greatly decline and then return to baseline after 2 days.^63^ Decreases may be associated with the increased ACTH secretions through signaling by the HPE axis.^13, 63^ Basophils also decrease 1 day after surgery and return to normal at day 5 and there is significant release in histamines.^64^ However, phenotypic changes to eosinophils and basophils following major surgeries are poorly documented.

### Changes in transcript level

Major trauma triggers the upregulation of different genes in local neutrophils at the local site. Genes involved include IL-1RA, IL-18 receptor-1, macrophage inflammatory protein-3*α*, macrophage migration inhibitory factor and group-II phospholipase-A2, whereas there is downregulation of IL-8 receptor-β.^65, 66^ Laudanski *et al.*^67^ also reported severe trauma-induced significant changes in the expression of 2800 genes in monocytes and >5500 genes in T lymphocytes, with an overall increased expression in >3000 genes in total white blood cells. Circulating monocytes also showed upregulation in Fas-Ligand, a member of TNF-superfamily.^68^ However, as mRNA expression alone cannot provide complete information on the levels and activities of the active molecules in the local site of trauma, mRNA levels need to be confirmed by analysis of the corresponding proteins.

### Changes in plasma biomarkers after sterile trauma

Complex changes in the levels of different cytokines occur after trauma. The most common changes are in IL-6. Miller *et al.*^69^ showed a post-traumatic decrease of IL-2, IFN-γ and IL-12 levels, whereas IL-10 and IL-4 levels were increased. This was associated with a shift from Th1 to Th2 T-cell responses. A comprehensive list of 109 investigations of changes in cytokine levels at different postoperative times is summarized in Table 2. Increased chemokines have important roles in the recruitment of mononuclear phagocytic cells from the bone marrow and spleen reservoirs to the site of injury.^70^ IL-8/CXCL-8 rises transiently following surgery, but drops back to baseline within few days. DAMPs, also called endogenous danger factors or alarmins, are a class of molecule that have vital roles in the recruitment of immune cells to the site of injury. DAMPs are released at the wound site during the trauma period and examples include HMGB-1, S100 proteins, heat-shock proteins and α-defensins.^71^ Routine arrays of DAMP concentrations in venous blood would help to better characterize post-traumatic immunity. Following blood loss, haemodilution, inflammation and other reasons, the levels of hemoglobin and hematocrit drop following major surgery.^72^ Complement activation produces complement components such as C5a and C3a that increase postoperatively.^73^ Release of histones and nucleosomes from damaged cells allows factor VII-activating protease to trigger the complement pathway and generation of C5a.^74^ Soluble CD-14, a marker of monocyte activation increases following surgery;^75^ whereas decreases are reported in soluble IL-6 receptor^76^ and soluble gp-130.^77^ In knee arthroplasty, Munoz *et al.*^59^ showed decreased levels of different immunoglobulins (IgG, IgA and IgM) at 6 h and these remained persistently low for at least 3 days.

Individuals do not respond equally to surgical trauma/stress due to variations in concentrations of soluble factors. This can be the result of genetic polymorphisms in the promoters of cytokine genes^78, 79^ as well as other factors such as race, age and obesity.^80, 81^ For this review, a comprehensive summary of published literature describing postoperative changes in the concentrations of different cytokines, chemokines and DAMP is presented in Table 2 and detailed in Supplementary Table S2. At the most frequently used time point, namely, 24 h, changes are summarized as follows:

- 87 out of 88 studies showed a consistent postoperative increase in IL-6; in one study IL-6 was undetectable.
- No increases were shown in 17% studies of IL-8, 30% studies of IL-10 and 27% studies of IL-1RA. But in the remainder studies all were increased.
- For IL-1β, IL-4, IL-12, IL-13, IL-17, IFN-γ, TNF-α and MCP-1, most studies showed no postoperative changes.
- Decreased IL-2 was shown in 54% (7/13) studies and unchanged in four studies.
- IL-5 and MIP-1α were unchanged in 50% of studies; although there were only four studies for IL-5 and 2 for MIP-1α.
- There was only one study of IL-22 and soluble IL-6 receptor each. IL-22 was increased, whereas soluble IL-6 receptor levels were down.
- For soluble CD-14, all three studies reported postoperative increases.
- HMGB-1 showed elevations in five studies; whereas one study showed no change.
- HSP-27 was studied and showed unchanged by one group. HSP-70, reported by two groups, also showed no postoperative changes. In the only study of HSP-60, levels were decreased.

Following trauma, necrotic cells release neo-antigens (by different enzymes and post-translational modifications) that trigger autoantibody IgM production by B1a B cells. A recent study showed that expression of Sialic acid-binding immunoglobulin-type lectin-10 (Siglec-10) by B1a B cells reduces their expansion and thus prevents the production of IgM.^82^ Thus, Siglec-10 may have a vital role in alleviating autoimmune reactions. Both systemic and local elevations of soluble CD24 and Siglec-10 were recorded following sterile trauma in our study.^83, 84^ This indicates a possible evolutionary advantage of PTI that PTI may suppresses auto-immunity to neo-antigens released from necrotic tissues at the sites of injury.^85^

### Effect of trauma on remote organs

#### Liver

The liver releases acute phase proteins (APPs) after trauma. IL-6 stimulates hepatocytes to release APPs such as C-reactive protein, serum amyloid-A (SAA), activated protein-C and alpha-1 antitrypsin.^86, 87, 88^ Elevated APPs stimulate the production of cytokine antagonists such as IL-1RA and soluble TNF receptors, thereby eventually resulting in immune-suppression and favoring wound healing.^89, 90^ Although APPs were initially thought to have only pro-inflammatory activities, recent studies suggest that their role following trauma is predominantly anti-inflammatory.^90, 91^

#### Gut

Sterile trauma also increases intestinal intercellular permeability of the patients. Maintenance of the gut epithelial barrier largely depends on the microbiota. On the basis of the degree of increases in intestinal permeability, sterile trauma may induce dysbiosis of the microbiome. This may trigger the translocation of bacteria from the gut to the circulation, and increase the risk of systemic PTI. However, whether PTI and increase gut permeability are directly associated with each other, remains to be investigated.^6, 7, 92^

**Figure 1:**
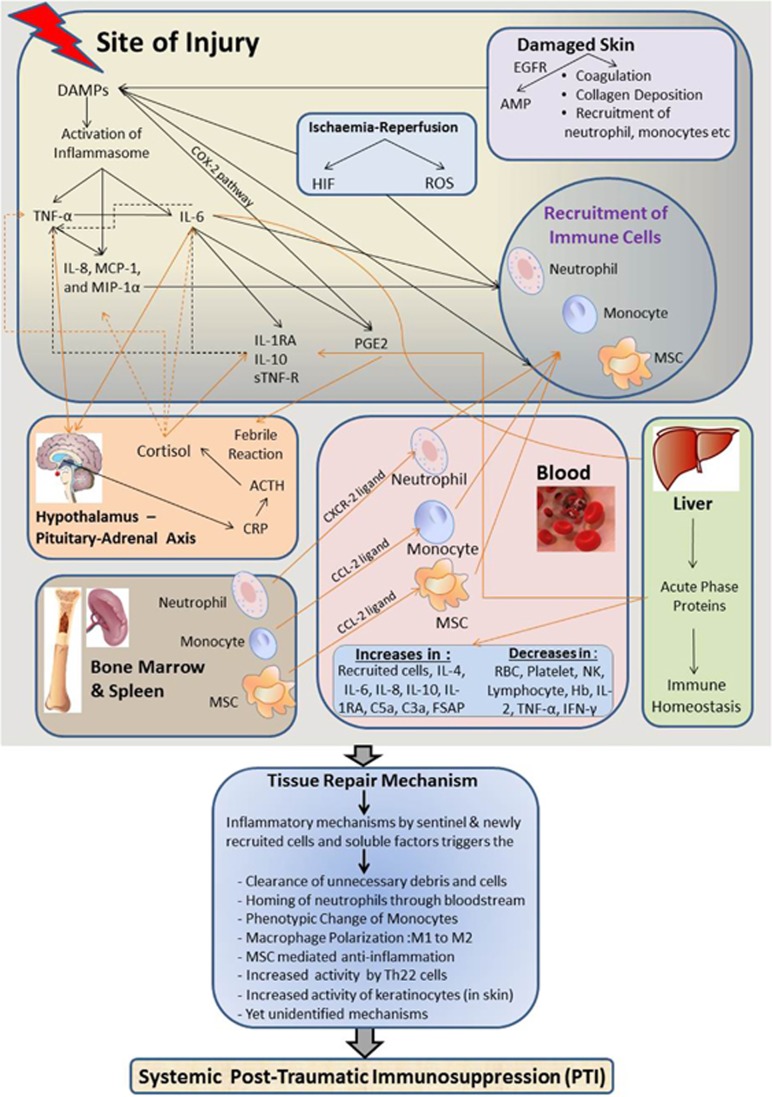
Schematic representation of PTI after sterile trauma. This figure schematically illustrates the sequence of events following major trauma. In brief, at the local injury site, release of different DAMPs by the damaged tissues induces viable cells to secrete chemokines such as IL-8, MCP-1 and MIP-1α, and the immediate secretion of inflammatory cytokines such as TNF-α. DAMPs release results in IL-6 and TNF-α production that activate the HPE axis to release ACTH, cortisol and also PGE2. These events then trigger the secretion of anti-inflammatory biomolecules such as IL-1RA, IL-10 and sTNF-R. Later, IL-6, by the virtue of its ability to trigger the release of acute phase proteins (APPs) by the liver, indirectly involved in reducing the inflammatory events at the injured site. In parallel with the immediate release of DAMPs, inflammatory cytokines and the activities of resident immune cells, there is recruitment of neutrophils, monocytes and MSCs from the blood to the injured site. Bone marrow and spleen act as reservoirs for the egression of these cells to the site of injury via the blood. By releasing antimicrobial peptides and helping in hemostasis, damaged skin also activates inflammasomes to release DAMPs at the local site. On the other hand, ischemia–reperfusion injury associated with the surgical procedure increases HIF and ROS expression that also trigger the production of DAMPs, and thus have an additional role in recruiting immune cells to the injured site. The ultimate goal of the above events following a major trauma is tissue remodeling and promotion of wound healing.

Figure 1 schematically represents an overview of these complex interactions and attempts to summarize the literature review undertaken. On the basis of the current understanding, Table 3 attempts to compare the key features of sterile trauma in the inflammation phase versus the immunosuppressive phase.

## Clinical consequences of PTI

Recent studies indicate that PTI following surgical trauma enhances patients' vulnerability to acquire exogenous infections from the hospital or endogenous infections associated with microbial translocations from the gut.^6, 7, 60, 93, 94, 95, 96^ Different forms of nosocomial infections may occur in the immuno-compromised patient in different body compartments.^97^ Respiratory tract infections are the most frequent, followed by urinary tract, wound and bloodstream infections.^98^ Furthermore, patients may acquire infections with multi-drug resistant bacteria that are resistant to most antibiotics.^99^ In extreme situations, PTI may eventually lead to severe sepsis, multiple organ failure and death. A rather alarming view is that the world is heading towards a post-antibiotic era due to the resistance of bacteria such as *Escherichia coli*, Klebsiella, tuberculosis and gonorrhea to all known antibiotics. This phenomenon has been termed the ‘antibiotic apocalypse' that may lead to life-threatening dangers even from a routine infection.^100^

Hospital-acquired pneumonia is the most common among all the nosocomial infections. Pneumonia although mostly caused by Gram-negative bacteria, can also manifest as infections by other bacteria and viruses. Multicentre studies on medical–surgical patients in intensive care units and on patients who acquired infections in intensive care unit revealed the highest episodes of pneumonia, most of which were ventilator associated.^98, 101^ Therefore, following surgery and the associated immune-compromised state, patients' vulnerability to acquired pneumonia increases.

The gastrointestinal tract is another important compartment in our body where infections are often reported following injuries such as blunt trauma and invasive abdominal surgeries. Infections in gastrointestinal tract also can be manifested with injuries to the spleen, pancreas and duodenum.^102, 103^

Urinary tract infection (UTI) mostly affects the bladder; however, it can also affect the kidney, ureters and urethra. UTI is another common infection in hospital caused by bacteria that enter the urethra and then the bladder. UTI is the second leading cause in intensive care units patients and it is mostly associated with catheterization.^98^ Major surgery most often requires the use of urinary catheters, therefore increasing the chance of UTI.

Bloodstream infections are also common nosocomial infections in hospital particularly from *Staphylococcus Aureus* and coagulase-negative *staphylococci*. This infection can spread into the bloodstream by several ways, especially via the intravenous line required in trauma patients.^104^

## Aggravating factors of PTI

PTI can be aggravated by many different biological factors. Blood transfusion, frequently a necessary step following surgery, also aggravates PTI. Transfusion of allogeneic blood or packed red blood cells aggravates PTI, as does transfusion of pre-deposited autologous blood.^61, 105, 106, 107^ Hemorrhage is another important aggravating factor that results in immunosuppression even without any major tissue trauma and may increase susceptibility to acquired infections.^108^ By releasing pro-inflammatory cytokines IFN-γ and IL-2 *in vitro* with enhanced production of IL-10 by T cells, hemorrhage may lead to PTI.^109^ Surgery itself is a major trigger of physical stress.^110^ In addition, factors such as pre-surgical medications and anesthesia show a direct association with the degree of surgical stresses.^111^ Other possible aggravating factors include mTBI, post-traumatic stress disorder and stroke.^29, 30, 60^ Use of anti-inflammatory/immunosuppressant drugs such as corticosteroids, cyclosporin-A and anti-TNFα treatments also depress patients' immunity and therefore may also increases susceptibility to acquire infections.^112, 113, 114^ Another biological factor aggravating PTI is immunesenescence. Elderly patients have weak immunity and are therefore more vulnerable to infections.^115, 116^ In animal studies, Zacks *et al.*^117^ previously showed relatively slower muscle regeneration and decreased phagocytic activities in older animals.

## Treatment of PTI

Considering all the above events following sterile trauma and the resulting immunosuppression, there has been increasing interest over the past decade in the use of immunostimulants to prevent PTI before, during or after surgical trauma. Commonly used immunostimulants can be either functional nutrients or immunotherapeutic agents.

Pre-existing malnutrition has a great impact on clinical outcome, and proper nutritional support helps to reverse PTI. Immuno-nutrients prescribed pre-, intra- and/or postoperatively can help to prevent post-surgical immunosuppression and include: glutamine, arginine, n-acetyl cysteine, branched-chain amino acids, glucan, nucleotides, long-chain n-3 fatty acids, antioxidant vitamins, trace elements and taurine.^118, 119, 120, 121, 122^ Patients treated with probiotics may also get substantial benefit following trauma.^123^

Strategies to modulate patients' immunity for better clinical outcome are mostly confined to drugs to prevent overwhelming inflammatory reactions following so-called SIRS. However, without knowing the underlying mechanisms of sepsis or sterile trauma, treatment with drugs may aggravate the clinical situation. This is reflected in the failure of many different clinical trials.^124, 125^ Immunotherapeutic agents used include: anti-PD1, mifamurtide, polyinosinic-polycytidylic acid, MF-59, imiquimod, luivac, myrrh, IDR-peptides, IL-7, rIFN-γ and IL-15.^125, 126, 127, 128^

Although allogeneic and pre-deposited autologous blood transfusion further suppressed PTI,^61^ thereby increasing the chance of nosocomial infections, we described the immunostimulatory activities of postoperatively salvaged autologous blood transfusion after major surgery.^61, 83^ This intriguing observation suggests that yet-to-be-identified natural bio-factors produced at the wound site may exist and if identified and administered intravenously, may have great therapeutic potentials to reverse PTI.

## Distinguishing sterile trauma from sepsis

One of the key unanswered questions at the present time in the field of inflammation research is how to distinguish PTI induced by sterile trauma from that induced by sepsis. The SIRS–CARS paradigm, although widely practiced by clinicians, does not distinguish these two conditions. In a recent seminal publication, Savage *et al.*^129^ analyzed the secretory capacity of glial cells stimulated with different DAMPs to mimic the sterile trauma condition) or the pathogen-associated molecular pattern molecule (lipopolysaccharide) to mimic sepsis. Differences were seen in the secretions of IL-1β, namely: elevations when stimulated by pathogen-associated molecular pattern, but unchanged when stimulated by DAMPs. However, secretion of IL-6 and CXCL-1 was identical in both the situations. This study thus highlighted the differences in cellular responses following sterile versus non-sterile activation and open new avenues to investigate the consequences of inflammasome activation on these processes.

Johnson *et al.*^78^ also showed significant variations in the gene expression profile of whole-blood cells taken from patients with sterile trauma versus early sepsis. Results obtained highlighted numerous genes that were preferentially increased in early sepsis. These unique genes could be subdivided into four broad categories, namely: innate immunity, cytokine receptors, T-helper cell differentiation and protein synthesis.^78^

Liu *et al.*^130^ distinguished certain differences between sterile trauma and sepsis. Thus, following sterile trauma, secreted DAMPs form tri-molecular complex with membrane bound CD24 molecules and the trans-membrane glycoprotein Siglec-10 that inhibits TLR/NLR-mediated inflammation. In contrast, in the case of microbial infections, the sialic acid chain of CD24 molecule is cleaved by the pathogen encoded sialidase enzyme; therefore the tri-molecular complex cannot form, resulting in the TLR/NLR-mediated inflammation.^130, 131, 132^ These *in vivo* mouse models and *in vitro* human studies need to be further validated in a clinical setting.

Both sterile and infectious trauma results in similar types of inflammation patterns, although a higher degree of inflammatory signals are recorded following sepsis compared with sterile trauma.^78^ This indicates that following trauma the human body responds by directing the injured organ to be healed by different mechanisms, whereas during sepsis these mechanisms may be altered due to continuous endotoxin (pathogen-associated molecular pattern)-induced inflammation.

## Conclusion

Sterile trauma involves both local and systemic responses representing a complex balance between pro- and anti-inflammatory mediators. In brief, at the trauma site, there is an immediate release of endogenous danger signals named DAMPs that increases local production of different cytokines, chemokines and other soluble factors by resident cells. Subsequently, via DAMPs and chemokines, there is further recruitment of neutrophils, monocytes and MSC into the local site of inflammation.^39, 133, 134, 135^ These newly recruited cells differentiate locally into cells secreting predominantly anti-inflammatory cytokines.^134, 136^ In addition, neutrophils and monocytes at the injured site are guided by DAMP-induced inflammasomes to have a role in apoptosis/necroptosis/NETosis and controlled clearing of damaged tissues.^137^ DAMPs also trigger the release of IL-6 that has an important role in the HPA axis to release steroids and in turn trigger the release of other immunosuppressive mediators to control inflammation at the wound site. IL-6 also induces hepatocytes to release APPs that mediate post-traumatic anti-inflammatory activities. All the cells and soluble factors at the local site contribute to tissue remodeling and wound healing.^1, 19^ These complex local and systemic changes following sterile trauma therefore lead to changes in patients' immune status that would vary between individuals.

Although many gaps in our understanding prevail, an outline is emerging of multiple mechanisms that restrain inflammation and suppress systemic immunity.^138^ Suppression of immunity in moments of crisis would appear to have conferred no evolutionary advantage to mammals, but without such restraints healing would be delayed by activation of tissue destructive inflammatory cascades.

**Figure 2:**
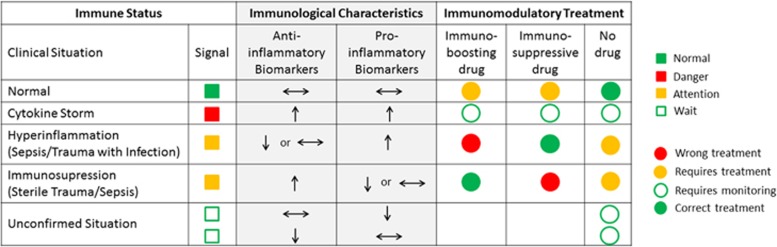
Measuring immune status helps in choosing appropriate immunomodulatory drug. Measuring a patient's immune status based on particular profile of specific pro- and anti-inflammatory proteins will help to indicate if the patient: (i) has a stable immune status and medication is not necessary, (ii) is at the stage of cytokine storm following trauma or infection when both pro- and anti-inflammatory cytokines rise, (iii) requires immediate attention with treatment (either immunostimulatory or immunosuppressive drug will be administered) or (iv) is not in need of medication but requires monitoring over the coming days. Knowing immune status will provide information following trauma or sepsis that can be monitored throughout the patient's recovery, thereby preventing the risk of unnecessary danger from incorrect therapy.

Sterile trauma leads to the changes in post-traumatic immunity and therefore a patient may be immune-compromised. Trauma accompanied by infection may also alter the immune status. Moreover, patients with these clinical conditions can be harmed, or put into unnecessary risk, by inappropriate treatment. Figure 2 describes the immune status following trauma and how inappropriate immunomodulatory drugs can result in aggravating a patient's outcome. Figure 2 also describes the benefit of knowing the immune status of patients to treat them with an appropriate immunomodulatory drug. Detailed analysis of immune status of patients therefore needs to be carried out to tailor the appropriate therapy. Measuring a patient's immune status individually before treatment would appear to be the best clinical approach to achieve the desired clinical outcome.

## Figures and Tables

**Table 1:** Lists of studies investigating the changes in biomarker levels at the surgical wound site

*Biomarker type*	*Biomarker name*	*No. of investigations*				*Total no. of studies*
		*Changes in wound site biomarker levels relative to pre-operative baseline levels*				
		*Decrease*	*No change*	*Increase*	*ND*	
Pro-inflammatory cytokines	IL-1β	—	2	10	1	13
	IL-2	—	1	—	1	2
	IL-6	—	—	24	—	24
	IL-12	—	1	—	—	1
	IL-17	—	1	—	—	1
	IFN-γ	—	1	—	—	1
	TNF-α	—	4	6	—	10
Anti-inflammatory cytokines	IL-4	—	1	2	—	3
	IL-5	—	1	—	—	1
	IL-10	1	1	3	—	5
	IL-13	—	1	—	—	1
	IL-1RA	—	—	2	—	2
Chemokines	IL-8	—	—	12	—	12
	MCP-1	—	—	1	—	1
	MIP-1α	—	1	—	—	1
Others	PGE2	—	—	2	—	2
	sIL-6R	1	—	—	—	1
	C3	—	—	7	—	7
	C5	—	—	4	—	4
	Platelet	4	—	4	—	8

Abbreviations: ND, not detectable; IL, interleukin; sIL-6R, soluble IL-6 receptor; TNF, tumor necrosis factor.

**Table 2:** Lists of investigations on changes in biomarker levels in postoperative venous blood following major surgery

*Biomarker type*	*Biomarker name*	*No. of investigations*				*Total no. of studies*
		*Postoperative changes (1 day) Also see* Supplementary Table S2				
		*Decrease*	*No change*	*Increase*	*ND*	
Pro-inflammatory cytokines	IL-1β	3	21	3	5	32
	IL-2	7	4	1	1	13
	IL-6	—	—	87	1	88
	IL-12	2	5	1	—	8
	IL-17	—	1	—	1	2
	IFN-γ	1	6	—	—	7
	TNF-α	5	23	6	5	39
	IL-22	—	—	1	—	1
Anti-inflammatory cytokines	IL-4	1	4	2	—	7
	IL-5	—	2	2	—	4
	IL-10	—	12	28	—	40
	IL-13	—	4	—	—	4
	IL-1RA	—	3	8	—	11
Chemokines	IL-8	—	7	31	2	40
	MCP-1	—	7	2	—	9
	MIP-1α	1	1	—	—	2
DAMPs	HMGB-1	—	1	5	—	6
	HSP-27	—	1	—	—	1
	HSP-60	1	—	—	—	1
	HSP-70	—	2	—	—	2
Others	sIL-6R	1	—	—	—	1
	sCD-14	—	—	3	—	3

Abbreviations: DAMP, damage-associated molecular pattern; IL, interleukin; IL-1RA, IL-1 receptor antagonist; ND, not detectable; sCD-14, soluble CD-14; sIL-6R, soluble IL-6 receptor; TNF, tumor necrosis factor.

**Table 3:** Different immunological phases in local site of trauma and systemic circulation following sterile trauma

	*Immunological events following sterile trauma*			
	*Activation phase*		*Suppression phase*	
	*Local*	*Systemic*	*Local*	*Systemic*
Macrophages	Inflammatory M1 polarization	—	Anti-inflammatory M2 polarization	Anti-inflammatory M2 polarization
Monocyte	Inflammatory phenotype	Monocytosis	Differentiation into anti-inflammatory phenotype	Increase in the number of anti-inflammatory phenotypes.
Neutrophil	Migration into the site of injury	Neutrophilia	Apoptosis of neutrophils at the site of injury	Overall increase in number of neutrophils however probably immature or less active
Lymphocyte	Inflammatory Th1 pathway	Increase in lymphocyte apoptosis	Anti-inflammatory Th2 pathway	Anti-inflammatory Th2 pathway
NK cells	—	—	Decrease in NK cell function	Decrease in NK cell function
DAMPs	Elevated levels of DAMPs trigger immediate release of cytokines to facilitate inflammation	—	Elevated levels of DAMPs direct anti-inflammatory activities to favor wound healing	DAMPs direct anti-inflammatory activities to favor systemic PTI
HPA axis	Immediate HPA axis-mediated signaling to initiate injury induced responses	—	Cortisol- and prostaglandin-mediated suppression to favor healing	Cortisol and prostaglandin also have role in systemic suppression
Acute phase proteins	Immediate release as part of body's inflammatory activities	Decreased levels of positive APPs	Trigger local immunosuppressive responses	Trigger immunosuppressive responses
MDSCs	—	Release of MDSCs from the source	Possible recruitment of MDSCs to the site of injury to facilitate local healing	Sustained release of MDSCs probably direct towards systemic suppression
Cytokines	Immediate release of different inflammatory and anti-inflammatory cytokines		Continued release of anti-inflammatory cytokines, whereas downregulation of pro-inflammatory cytokine release	Reductions in inflammatory cytokines such as IL-1β, IL-2, TNF-α, IFN-γ and IL-17A, whereas elevations in anti-inflammatory IL-5

Abbreviations: AAP, acute phase protein; DAMP, damage-associated molecular pattern; IL, interleukin; HPA, hypothalamus–pituitary–adrenal; MDSC, myeloid-derived suppressor cell; NK, natural killer; TNF, tumor necrosis factor.
