# Supplementary material for: Sterile post-traumatic immunosuppression
Source: Clin Transl Immunology. 2016 Apr 29;5(4):e77–. doi: 10.1038/cti.2016.13 (PMC4855263; doi:10.1038/cti.2016.13)
Supplement: Supplementary Information [file cti201613x1.pdf]

SUPPLEMENTARY INFORMATION

Sterile Post-traumatic Immunosuppression

Md Nahidul Islam, M.Sc., Ph.D. [1], Benjamin A Bradley, MBChB, Ph.D. [1,2], Rhodri Ceredig, F.R.C.P., Ph.D. [1]

[1] Regenerative Medicine Institute, National Centre of Biomedical Engineering Science and School of Medicine, Nursing and Health Sciences, National University of Ireland, Galway, Galway, Ireland.
[2] Orthopaedic Research Unit, Learning and Research Building, Southmead Hospital, University of Bristol, Bristol, United Kingdom.

Contents

Page 2: Supplementary Table S1. Levels of Biomarkers in Wound Blood

Page 3-6: Supplementary Table S2. Levels of Biomarkers Postoperatively Following Major Surgery

Page 7-16: References for Supplementary Tables

Supplementary Table S1 : Levels of Biomarkers in Wound Blood

Type of Surgery	Collection Period	C3	C5	IL-1β	IL-2	IL-4	IL-5	IL-6	IL-8	IL-10	IL-12	IL-13	IL-17	IL-1RA	TNF-α	IFN-γ	MCP-1	MIP-1α	PGE2	sIL-6R	Platelet count	References
AAA	post	INC		INC				INC	INC						INC							1
THA	post	INC		INC				INC	INC						INC							1
THA	intra	INC	INC	N.D.				INC	INC						N.C.							2
THA	intra	INC						INC	INC						INC							3
artery bypass	post	INC	INC	INC				INC	INC						INC							4
CPB	post	INC		INC				INC		INC												5
TKA	post	INC																				6
TKA, THA	post		INC					INC	INC													7
Spine surgery	post			INC				INC														8
Thoracic scoliosis	post			INC	N.D.			INC		INC				INC	N.C.					DEC		9
THA	post			INC		INC		INC	INC													10
TKA, THA	post			INC		INC		INC	INC													11
THA	post			INC				INC	INC						INC							12
TKA	post			INC				INC														13
THA	post			N.C.				INC		DEC					N.C.							14
THA	post			N.C.	N.C.	N.C.	N.C.	INC	INC	N.C.	N.C.	N.C.	N.C.	INC								15
CABG	post							INC														16
CPB	post							INC														17
Thoracic scoliosis	post							INC														18
TKA	post							INC		INC					INC							19
TKA	post							INC														20
TKA, THA	post							INC														21
TKA	post							INC							N.C.							22
TKA	post							INC														23
TKA	post							INC	INC													24
THA	post								INC							N.C.	INC	N.C.				25
Thoracic scoliosis	post		INC																			26
CPB	post																		INC			27
THA	post																		INC			28
surgery	post																				DEC	29
Cardiac surgery	post																				DEC	30
TKA, THA	post																				DEC	31
TKA, THA	post																				DEC	32
Cardiac surgery	post																				INC	33
aortic surgery	intra																				INC	34
CPB	post																				INC	35
aortic surgery	intra																				INC	36

Here AAA = Abdominal aortic aneurysm, THA = Total Hip Arthroplasty, TKA = Total Knee Arthroplasty, CABG = Coronary Artery Bypass Grafting, CPB = Cardio Pulmonary Bypass, h = hour. C = Complement; IL-(1β) = Interleukin-(1-beta); IL-1RA = IL-1 Receptor Antagonist; TNF = Tumour Necrosis Factor; IFN = Interferon; MCP = Monocyte Chemotactic Protein; MIP = Macrophage Inflammatory Protein; PGE2 = Prostaglandin E2; sIL-6R = Soluble IL-6 Receptor.
INC = increase, DEC = Decrease, N.C. = No Change, N.D. = Not Detectable

Supplementary Table S2 : Levels of Biomarkers Postoperatively Following Major Surgery

Surgery Type	No. of Patients	Postop days	IL-1β	IL-2	IL-4	IL-5	IL-6	IL-8	IL-10	IL-12	IL-13	IL-17	IL-22	IL-1RA	IFN-γ	TNF-α	MCP-1	MIP-1α	sCD-14	sIL-6R	HMGB-1	HSP-27	HSP-60	HSP-70	References
CPB with SIRS	12	1d, 2d	DEC				INC	N.C.																	37
primary breast cancer surgery	32		DEC																						38
CPB	30	1 d, 2 d	DEC	DEC			INC	INC	INC							**DEC**									39
reconstructive surgery	14	6 h	INC	N.C.			INC																		40
CABG	13	after surgery	INC				INC	INC																	41
THA	30	1	INC	DEC			INC		INC																42
CPB		4h, 1 d	INC at 4h				INC																		43
TKA	20	1, 6, 24, and 48 h	N.C.				INC																		13
CABG	32	1 d	N.C.				INC									**N.C.**									44
Abdominal Surgery		1d, 2d, 3d	N.C.				INC	INC																	45
Coronary stenting		1,2,6 h	N.C.				INC									**N.C.**									46
THA	7	1, 3 d	N.C.				INC		N.C.										INC						47
TKA		3 days	N.C.				INC									**N.C.**									48
CABG with CPB	78	24 h till 3 d	N.C.				INC	INC								**N.C.**									49
CABG	10	3, 6, and 24 h	N.C.				INC	N.C.								**N.C.**									50
TJA	10	4 h	N.C.				INC		N.C.							**N.C.**									14
CPB		1,2,3, d etc	N.C.				INC		INC 1d							**N.C.**									51
pyeloplasty	31	1d, 2d	N.C.				INC	N.C.	N.C.							**N.C.**									52
TKA	85	2,3,5	N.C.				INC	N.C.	N.C.							**N.C.**									53
cardiac surgery	57	1 d	N.C.				INC	INC		INC					N.C.										54
Pancreatico-duodenectomy	22	1d, 3d	N.C.				INC	INC	INC 1d					INC		**N.C. 1d, DEC 3d**									55
THA	13	3 days	N.C.				INC	N.C.	N.C.	N.C.						**N.C.**									56
open thoracic surgery	63	1d	N.C.				INC	N.C.	N.C.	N.C.						**N.C.**									57
cardiac surgery	274	day of op, 1 d	N.C.	N.C.	N.C.	N.C.	INC	INC	INC op. day, N.C. 1d							**N.C.**									58
CPB	38	1d	N.C.				INC	INC	INC	N.C.					N.C.		N.C.	N.C.							59
THA	7	1d	N.C.	N.C.	N.C.	N.C.	INC	N.C.	N.C.	N.C.	N.C.	N.C.		N.C.											15
TKA	35	24h (next morning)	N.C.	N.C.	N.C.	INC	INC	INC	INC	N.C.	N.C.				N.C.	**DEC**									60
Cardiac	24	1, 3 d	N.D.													**N.D.**									61
Lung transplantation	20	1,2,3,4 d etc	N.D.				N.D.	N.D.																	62

Here THA = Total Hip Arthroplasty, TKA = Total Knee Arthroplasty, TJA = Total Joint Arthroplasty, CABG = Coronary Artery Bypass Grafting, CPB = Cardio Pulmonary Bypass; D = Day, h = hour. IL-(1β) = Interleukin-(1-beta); IL-1RA = IL-1 Receptor Antagonist; IFN = Interferon; TNF = Tumour Necrosis Factor; MCP = Monocyte Chemotactic Protein; MIP = Macrophage Inflammatory Protein; HMGB = High Mobility Group Protein; INC = increase, DEC = Decrease, N.C. = No Change, N.D. = Not Detectable

Surgery Type	No. of Patients	Postop days	IL-1β	IL-2	IL-4	IL-5	IL-6	IL-8	IL-10	IL-12	IL-13	IL-17	IL-22	IL-1RA	IFN-γ	TNF-α	MCP-1	MIP-1α	sCD-14	sIL-6R	HMGB-1	HSP-27	HSP-60	HSP-70	References
CABG	12	20 min, 1 d	N.D.				INC		INC							**N.D.**									63
cardiac surgery	19	after surgery	N.D.				INC									**N.D.**					N.C.				64
heart valve replacement	10	1d, 3d, 7d		DEC																					65
Reperfusion Injury				DEC																					66
CABG	24	1 d		DEC						DEC at 6h															67
CABG / CPB	20	2 d		DEC			INC	INC																	68
thoracotomy	47	1d, 2d		DEC	DEC		INC		INC							**DEC**									69
CABG	250	1 d		INC																					70
CABG	15	1d	N.D.	N.D.			INC	INC	N.C.					N.C.		**N.D./ N.C.**									71
CPB	16	16 h			INC										N.C.										72
gastric resection or gastrectomy		3h			INC		INC		INC																73
CABG	16	1 d			N.C.		INC	INC	N.C.						N.C.										74
cardiac surgery	25	1d, 3d, 5d				INC 3d, 5d	INC 1d, N.C. 5d		INC 1d, N.C. 5d						N.C.	**INC 1d, N.C. 5d**									75
THA		2 days					INC																		76
open heart surgery		every hour for few days					INC																		77
THA	10	24 h					INC													DEC					78
CABG	20						INC																		79
TJA	30	1 h					INC																		80
abdominal and thoraco-abdominal surgery	18	1d, 3d					INC																		81
TKA	81	6 h					INC																		82
THA							INC																		83
CPB	21	1d					INC																		84
thoracic surgery	29	1d					INC																		85
major surgery for colon cancer		3h, 1 d					INC																		86
TKA	49	1 d					INC																		87
Pneumonectomy	46	1d					INC																		88
cardiac surgery	12	1d					INC									**N.C. /N.D.**									89
Diff Surgery		1d, 2d					INC	INC																	90
CABG	15	1 d					INC									**N.C.**									91
major operations	28	36h					INC									**N.D.**									92

Here THA = Total Hip Arthroplasty, TKA = Total Knee Arthroplasty, TJA = Total Joint Arthroplasty, CABG = Coronary Artery Bypass Grafting, CPB = Cardio Pulmonary Bypass; D = Day, h = hour. IL-(1β) = Interleukin-(1-beta); IL-1RA = IL-1 Receptor Antagonist; IFN = Interferon; TNF = Tumour Necrosis Factor; MCP = Monocyte Chemotactic Protein; MIP = Macrophage Inflammatory Protein; HMGB = High Mobility Group Protein; INC = increase, DEC = Decrease, N.C. = No Change, N.D. = Not Detectable

Surgery Type	No. of Patients	Postop days	IL-1β	IL-2	IL-4	IL-5	IL-6	IL-8	IL-10	IL-12	IL-13	IL-17	IL-22	IL-1RA	IFN-γ	TNF-α	MCP-1	MIP-1α	sCD-14	sIL-6R	HMGB-1	HSP-27	HSP-60	HSP-70	References
TJA	13						INC	INC																	11
CPB	18	1d					INC										N.C.								93
THA	12	4 h					INC									**N.C.**									94
cardiac surgery	30	1d, 3d					INC	N.C./INC																	95
cardiac surgery	108	1d, 3d					INC	INC																	96
cardiac surgery	93	1d					INC										N.C.								97
Abdominal hysterectomy		1d					INC		N.C.																98
laparoscopy and laparotomy	40(25/15)	1d, 2d					INC		INC 1d																99
lung cancer surgery	27	1d, 3d					INC							INC											100
major lung resection	33	1d, 3d					INC							N.C.											101
TKA	20	12h, 1d, 2d					INC									**N.C.**									22
TKA	11	36 h					INC	INC																	24
CPB	44	2d					INC	INC																	102
CPB	250	1d					INC									**INC**									103
esophagectomy for squamous cell carcinoma	11	1d					INC	INC																	104
laparoscopic cholecystectomy	17	1d, 2d					INC		INC																105
CPB	20	6h, 18 h					INC		INC																106
radical prostatectomy	165	1d					INC		INC																107
femoral nailing	59	1d, 2d					INC	INC	INC																108
esophagectomy	20	postop					INC	INC	INC																109
CPB	16	1d					INC		INC							**N.C.**									110
major abdominal surgery	26	12h, 3d					INC		INC 12h							**N.C.**									111
cardiac surgery	15	1d, 2d, 5d					INC									**N.C.**	N.C.								112
CABG	44	1d, 2d					INC	INC								**N.C.**									113
cardiac surgery	92	3d					INC	INC						INC											114
TKA	9	4h					INC		N.C.							**N.C.**									23
esophagectomy	29	1d					INC		INC					INC											115
CPB	25	1d, 2d					INC		INC				INC			**INC**									116
spine surgery	10	1d, 3d					INC	INC	INC					INC											117
abdominal surgery	40	1d, 2d					INC	INC 1d	INC							**INC 1d**									118
cardiac surgery	15	1d, 2d					INC	INC 1d, N.C. 2d								**N.C.**	INC 1d, N.C. 2d								119

Here THA = Total Hip Arthroplasty, TKA = Total Knee Arthroplasty, TJA = Total Joint Arthroplasty, CABG = Coronary Artery Bypass Grafting, CPB = Cardio Pulmonary Bypass; D = Day, h = hour. IL-(1β) = Interleukin-(1-beta); IL-1RA = IL-1 Receptor Antagonist; IFN = Interferon; TNF = Tumour Necrosis Factor; MCP = Monocyte Chemotactic Protein; MIP = Macrophage Inflammatory Protein; HMGB = High Mobility Group Protein; INC = increase, DEC = Decrease, N.C. = No Change, N.D. = Not Detectable

Surgery Type	No. of Patients	Postop days	IL-1β	IL-2	IL-4	IL-5	IL-6	IL-8	IL-10	IL-12	IL-13	IL-17	IL-22	IL-1RA	IFN-γ	TNF-α	MCP-1	MIP-1α	sCD-14	sIL-6R	HMGB-1	HSP-27	HSP-60	HSP-70	References
valvular heart surgery	24	1d, 2d					INC	INC	INC							**INC**									120
CABG	54	1d, 3d, 7d					INC										N.C.								121
CABG with CPB	20	1d						INC	INC							**INC**									122
CABG without CPB	8							INC	INC					INC											123
CPB	24	1 d						N.C.																	124
THA	7	4h, 1 d						N.D.							DEC		INC 4h, N.C.1 d	DEC							25
cardiac surgery	92	3d							INC																114
thoracic scoliosis	8	1, 2, 4, and 6 h							INC					INC											125
abdominal aortic surgery	37	1 d								DEC															126
CABG	30	1d									N.C.														127
cardiac surgery	34	1 d									N.C.														128
cataract surgery		1d										N.D.													129
orthotopic heart transplantation		2d, 5d												INC											130
Operation																**DEC**									131
CABG	20	1d															INC		INC						132
CPB	40	1d																	INC						133
transthoracic esophagectomy	24	1d, 2d																			INC				134
blunt trauma	20	3d																			INC				135
gastrointestinal surgery	28	3d																			INC				136
General Elective Surgery	37	3d																			INC				137
thoracic aortic aneurysm (TAA) repair	20	2d																			INC				138
CABG	40	1d																				N.C.	DEC	N.C.	139
CABG	8	1h, 1d																						INC	140
CABG	20	19h, 43h																						N.C.	141

Here THA = Total Hip Arthroplasty, TKA = Total Knee Arthroplasty, TJA = Total Joint Arthroplasty, CABG = Coronary Artery Bypass Grafting, CPB = Cardio Pulmonary Bypass; D = Day, h = hour. IL-(1β) = Interleukin-(1-beta); IL-1RA = IL-1 Receptor Antagonist; IFN = Interferon; TNF = Tumour Necrosis Factor; MCP = Monocyte Chemotactic Protein; MIP = Macrophage Inflammatory Protein; HMGB = High Mobility Group Protein; INC = increase, DEC = Decrease, N.C. = No Change, N.D. = Not Detectable

References for Supplementary Tables:

1. Lindholm E, Seljeflot I, Aune E, Kirkeboen KA. Proinflammatory cytokines and complement activation in salvaged blood from abdominal aortic aneurism surgery and total hip replacement surgery. *Transfusion (Paris)* 2012; **52**: 1761-1769.
2. Kvarnstrom A, Schmidt A, Tylman M, Jacobsson M, Bengtsson A. Complement split products and proinflammatory cytokines in intraoperatively salvaged unwashed blood during hip replacement: comparison between heparin-coated and non-heparin-coated autotransfusion systems. *Vox Sang* 2008; **95**: 33-38.
3. Stachura A, Krol R, Poplawski T, Michalik D, Pomianowski S, Jacobsson M, et al. Transfusion of intra-operative autologous whole blood: influence on complement activation and interleukin formation. *Vox Sang* 2011; **100**: 239-246.
4. Schonberger JP, van Oeveren W, Bredee JJ, Everts PA, de Haan J, Wildevuur CR. Systemic blood activation during and after autotransfusion. *Ann Thorac Surg* 1994; **57**: 1256-1262.
5. Salas Millan J, de Vega N, Carmona Aurioles J, Negri Arjona S, Garcia-Vallejo J, Munoz Gomez M. [Autotransfusion after cardiac surgery. Hematological, biochemical and immunological properties of shed mediastinal blood]. *Rev Esp Anestesiol Reanim* 2001; **48**: 122-130.
6. Jensen CM, Pilegaard R, Hviid K, Nielsen JD, Nielsen HJ. Quality of reinfused drainage blood after total knee arthroplasty. *J Arthroplasty* 1999; **14**: 312-318.
7. Andersson I, Tylman M, Bengtson JP, Bengtsson A. Complement split products and pro-inflammatory cytokines in salvaged blood after hip and knee arthroplasty. *Canadian Journal of Anesthesia* 2001; **48**: 251-255.
8. Sebastian C, Romero R, Olalla E, Ferrer C, Garcia-Vallejo JJ, Munoz M. Postoperative blood salvage and reinfusion in spinal surgery: blood quality, effectiveness and impact on patient blood parameters. *Eur Spine J* 2000; **9**: 458-465.
9. Krohn CD, Reikerås O, Bjørnsen S, Brosstad F. Fibrinolytic activity and postoperative salvaged untreated blood for autologous transfusion in major orthopaedic surgery. *Eur J Surg* 2001; **167**: 168-172.
10. Arnestad JP, Bengtsson A, Bengtson JP, Tylman M, Redl H, Schlag G. Formation of cytokines by retransfusion of shed whole blood. *British Journal of Anaesthesia* 1994; **72**: 422-425.

11. Southern EP, Huo MH, Mehta JR, Keggi KJ. Unwashed wound drainage blood. What are we giving our patients? *Clin Orthop Relat Res* 1995; 235-246.
12. Witt M, Claus R, Burstein C, Anders O. TISSUE FACTOR, CYTOKINES, AND SOLUBLE CELL SURFACE RECEPTORS IN RETRANSFUSED UNWASHED SHED BLOOD AND IN SERUM IN TOTAL HIP REPLACEMENT. *Journal of Bone and Joint Surgery, British Volume* 2002; **84-B**: 22-b-22.
13. Andres BM, Taub DD, Gurkan I, Wenz JF. Postoperative fever after total knee arthroplasty: the role of cytokines. *Clin Orthop Relat Res* 2003; 221-231.
14. Clementsen T, Krohn CD, Reikeras O. Systemic and local cytokine patterns during total hip surgery. *Scand J Clin Lab Invest* 2006; **66**: 535-542.

15. Bastian D, Tamburstuen MV, Lyngstadaas SP, Reikeras O. Systemic and local cytokine kinetics after total hip replacement surgery. *Eur Surg Res* 2008; **41**: 334-340.
16. Schmidt H. Autotransfusion of mediastinal blood after coronary artery bypass grafting. *Dan Med Bull* 2000; **47**: 79-93.
17. Schmidt H, Bendtzen K, Mortensen PE. The inflammatory cytokine response after autotransfusion of shed mediastinal blood. *Acta Anaesthesiol Scand* 1998; **42**: 558-564.
18. Krohn CD, Reikeras O, Bjornsen S, Brosstad F. Tissue factor antigen and activity in serum of postoperatively shed blood used for autologous transfusion. *Blood Coagul Fibrinolysis* 2000; **11**: 219-223.
19. Bentzien F, Brand JM, Rohrs E, Munkel H, Schmucker P. [Mechanical autotransfusion procedures. The effect cytokines and leukocytes on washed erythrocyte concentrate]. *Anaesthesist* 2000; **49**: 505-510.
20. Handel M, Winkler J, Hornlein RF, Northoff H, Heeg P, Sell S. Time-related changes of collected shed blood in autologous retransfusion after total knee arthroplasty. *Arch Orthop Trauma Surg* 2001; **121**: 557-560.
21. Kristiansson M, Soop M, Saraste L, Sundqvist KG, Suontaka AM, Blomback M. Cytokine and coagulation characteristics of retrieved blood after arthroplasty. *Intensive Care Med* 1995; **21**: 989-995.
22. Feng Y, Ju H, Yang B, An H. Effects of a selective cyclooxygenase-2 inhibitor on postoperative inflammatory reaction and pain after total knee replacement. *J Pain* 2008; **9**: 45-52.
23. Clementsen T, Reikeras O. Cytokine patterns after tourniquet-induced skeletal muscle ischaemia reperfusion in total knee replacement. *Scand J Clin Lab Invest* 2008; **68**: 154-159.
24. Lisowska B, Maslinski W, Maldyk P, Zabek J, Baranowska E. The role of cytokines in inflammatory response after total knee arthroplasty in patients with rheumatoid arthritis. *Rheumatol Int* 2008; **28**: 667-671.
25. Bastian D, Tamburstuen MV, Lyngstadaas SP, Reikeras O. Local and systemic chemokine patterns in a human musculoskeletal trauma model. *Inflamm Res* 2009; **58**: 483-489.
26. Krohn CD, Reikerås O, Aasen AO. The cytokines IL-1β and IL-1 receptor antagonist, IL-2 and IL-2 soluble receptor-α, IL-6 and IL-6 soluble receptor, TNF-α and TNF soluble receptor I, and IL10 in drained and systemic blood after major orthopaedic surgery. *Eur J Surg* 1999; **165**: 101-109.
27. Sandoval S, Alrawi S, Samee M, Satheesan R, Raju R, Cunningham JN, et al. A cytokine analysis of the effect of cell saver on blood in coronary bypass surgery. *Heart Surg Forum* 2001; **4**: 113-117; discussion 117-119.
28. Mottl-Link S, Russlies M, Klinger M, Seyfarth M, Ascherl R, Gradinger R. Erythrocytes and proinflammatory mediators in wound drainage. *Vox Sang* 1998; **75**: 205-211.
29. Hartz RS, Smith JA, Green D. Autotransfusion after cardiac operation. Assessment of hemostatic factors. *J Thorac Cardiovasc Surg* 1988; **96**: 178-182.
30. Axford TC, Dearani JA, Ragno G, MacGregor H, Patel MA, Valeri CR, et al. Safety and therapeutic effectiveness of reinfused shed blood after open heart surgery. *Ann Thorac Surg* 1994; **57**: 615-622.

31. Duchow J, Ames M, Hess T, Seyfert U. Activation of plasma coagulation by retransfusion of unwashed drainage blood after hip joint arthroplasty: a prospective study. *J Arthroplasty* 2001; **16**: 844-849.
32. Wixson RL, Kwaan HC, Spies SM, Zimmer AM. Reinfusion of postoperative wound drainage in total joint arthroplasty. Red blood cell survival and coagulopathy risk. *J Arthroplasty* 1994; **9**: 351-358.
33. Schaff HV, Hauer JM, Bell WR, Gardner TJ, Donahoo JS, Gott VL, et al. Autotransfusion of shed mediastinal blood after cardiac surgery: a prospective study. *J Thorac Cardiovasc Surg* 1978; **75**: 632-641.
34. Lawrence-Brown MM, Couch C, Halliday M, Hellings M, Barr A, Marshall L. D-dimer levels in blood salvage for autotransfusion. *Aust N Z J Surg* 1989; **59**: 67-70.
35. de Haan J, Schonberger J, Haan J, van Oeveren W, Eijgelaar A. Tissue-type plasminogen activator and fibrin monomers synergistically cause platelet dysfunction during retransfusion of shed blood after cardiopulmonary bypass. *J Thorac Cardiovasc Surg* 1993; **106**: 1017-1023.
36. Bartels C, Bechtel JV, Winkler C, Horsch S. Intraoperative autotransfusion in aortic surgery: comparison of whole blood autotransfusion versus cell separation. *J Vasc Surg* 1996; **24**: 102-108.
37. Sablotzki A, Friedrich I, Muhling J, Dehne MG, Spillner J, Silber RE, et al. The systemic inflammatory response syndrome following cardiac surgery: different expression of proinflammatory cytokines and procalcitonin in patients with and without multiorgan dysfunctions. *Perfusion* 2002; **17**: 103-109.
38. Deegan CA, Murray D, Doran P, Moriarty DC, Sessler DI, Mascha E, et al. Anesthetic technique and the cytokine and matrix metalloproteinase response to primary breast cancer surgery. *Reg Anesth Pain Med* 2010; **35**: 490-495.
39. Presta P, Rubino AS, Lucisano G, Serraino GF, Foti D, Gulletta E, et al. Preoperative statins improve recovery of renal function but not by an anti-inflammatory effect: observational study in 69 elderly patients undergoing cardiac surgery. *Int Urol Nephrol* 2011; **43**: 601-609.
40. Hayward AL, Deehan DJ, Aspden RM, Sutherland AG. Analysis of sequential cytokine release after ACL reconstruction. *Knee Surg Sports Traumatol Arthrosc* 2011; **19**: 1709-1715.
41. Machado LB, Negri EM, Bonafe WW, Santos LM, Sa Malbouisson LM, Carmona MJ. [Evaluation of cytokine levels and pulmonary function in patients undergoing coronary artery bypass graft]. *Rev Bras Anestesiol* 2011; **61**: 275-285.
42. Mei XL, Guo T, Zhao JN. [Clinical significance and expression of the inflammatory cytokines (IL-1, IL-2, IL-6 and IL-10) in blood serum of the patients after total hip replacement]. *Zhongguo Gu Shang* 2011; **24**: 463-465.
43. Ferroni P, Speziale G, Ruvolo G, Giovannelli A, Pulcinelli FM, Lenti L, et al. Platelet activation and cytokine production during hypothermic cardiopulmonary bypass--a possible correlation? *J Thromb Haemost* 1998; **80**: 58-64.
44. Shimamoto A, Kanemitsu S, Fujinaga K, Takao M, Onoda K, Shimono T, et al. Biocompatibility of silicone-coated oxygenator in cardiopulmonary bypass. *Ann Thorac Surg* 2000; **69**: 115-120.
45. Chachkhiani I, Gurlich R, Maruna P, Frasko R, Lindner J. The postoperative stress response and its reflection in cytokine network and leptin plasma levels. *Physiol Res* 2005; **54**: 279-285.
46. ElMokhtari N, Zschernitz S, Sebens S, Simon-Herrmann G, Kruger D. Cardiac release and kinetics of cytokines after elective bare metal coronary stenting. *J Thromb Thrombolysis* 2010; **30**: 391-397.

47. Bastian D, Tamburstuen MV, Lyngstadaas SP, Reikeras O. LBP and sCD14 patterns in total hip replacement surgery performed during combined spinal/epidural anaesthesia. *Scand J Clin Lab Invest* 2011; **71**: 486-491.
48. Notarnicola A, Moretti L, Tafuri S, Vacca A, Marella G, Moretti B. Postoperative pain monitor after total knee replacement. *Musculoskelet Surg* 2011; **95**: 19-24.
49. Rothenburger M, Soeparwata R, Deng MC, Schmid C, Berendes E, Tjan TD, et al. Prediction of clinical outcome after cardiac surgery: the role of cytokines, endotoxin, and anti-endotoxin core antibodies. *Shock* 2001; **16 Suppl 1**: 44-50.
50. Anic D, Gasparovic H, Ivancan V, Batinic D. Effects of corticosteroids on inflammatory response following cardiopulmonary bypass. *Croat Med J* 2004; **45**: 158-161.
51. Poulsen TD, Andersen LW, Steinbruchel D, Gotze JP, Jorgensen OS, Olsen NV. Two large preoperative doses of erythropoietin do not reduce the systemic inflammatory response to cardiac surgery. *J Cardiothorac Vasc Anesth* 2009; **23**: 316-323.
52. Wang L, Qin W, Tian F, Zhang G, Yuan J, Wang H. Cytokine responses following laparoscopic or open pyeloplasty in children. *Surg Endosc* 2009; **23**: 544-549.
53. Drosos GI, Blatsoukas KS, Ververidis A, Tripsianis G, Chloropoulou P, Iatrou C, et al. Blood transfusion and cytokines' changes in total knee replacement. *Arch Orthop Trauma Surg* 2012; **132**: 1505-1513.
54. Prieto MA, Guash S, Mendez JC, Munoz C, Planas A, Reyes G. Does use of cell saver decrease the inflammatory response in cardiac surgery? *Asian Cardiovascular and Thoracic Annals* 2013; **21**: 37-42.
55. Slotwinski R, Olszewski WL, Slotkowski M, Lech G, Zaleska M, Slotwinska SM, et al. Can the interleukin-1 receptor antagonist (IL-1ra) be a marker of anti-inflammatory response to enteral immunonutrition in malnourished patients after pancreaticoduodenectomy? *JOP* 2007; **8**: 759-769.
56. Bjornsson GL, Thorsteinsson L, Gudmundsson KO, Jonsson H, Jr., Gudmundsson S, Gudbjornsson B. Inflammatory cytokines in relation to adrenal response following total hip replacement. *Scand Journal of Immunology* 2007; **65**: 99-105.
57. Schilling T, Kozian A, Senturk M, Huth C, Reinhold A, Hedenstierna G, et al. Effects of volatile and intravenous anesthesia on the alveolar and systemic inflammatory response in thoracic surgical patients. *Anesthesiology* 2011; **115**: 65-74.
58. Koch A, Hamann L, Schott M, Boehm O, Grotemeyer D, Kurt M, et al. Genetic variation of TLR4 influences immunoendocrine stress response: an observational study in cardiac surgical patients. *Critical Care* 2011; **15**: R109.
59. Kim T, Arnaoutakis GJ, Bihorac A, Martin TD, Hess PJ, Jr., Klodell CT, et al. Early blood biomarkers predict organ injury and resource utilization following complex cardiac surgery. *J Surg Res* 2011; **168**: 168-172.
60. Bromander S, Anckarsater R, Kristiansson M, Blennow K, Zetterberg H, Anckarsater H, et al. Changes in serum and cerebrospinal fluid cytokines in response to non-neurological surgery: an observational study. *J Neuroinflammation* 2012; **9**: 242.
61. Furunaga A, Tsuboi H, Sasaki J, Itoh H, Mikamo A, Okada H, et al. [Influence of cardiopulmonary bypass on biological response]. *Kyobu Geka* 1995; **48**: 1085-1087.
62. Teixeira RH, Antonangelo L, Vargas FS, Caramori ML, Afonso JE, Jr., Acencio MM, et al. Cytokine profile in pleural fluid and serum after lung transplantation. *Transplant Proc* 2010; **42**: 531-534.

63. Kleinschmidt S, Wanner GA, Bussmann D, Kremer JP, Ziegenfuss T, Menger MD, et al. Proinflammatory cytokine gene expression in whole blood from patients undergoing coronary artery bypass surgery and its modulation by pentoxifylline. *Shock* 1998; **9**: 12-20.
64. Haque A, Kunimoto F, Narahara H, Okawa M, Hinohara H, Kurabayashi M, et al. High mobility group box 1 levels in on and off-pump cardiac surgery patients. *Int Heart J* 2011; **52**: 170-174.
65. Luo J, Wang D, Lan H, Sun Z. Effects of aprotinin on serum interleukin-2 and soluble interleukin-2 receptor during cardiopulmonary bypass. *J Tongji Med Univ* 2000; **20**: 143-144.
66. Sullivan PJ, Sweeney KJ, Hirpara KM, Malone CB, Curtin W, Kerin MJ. Cyclical ischaemic preconditioning modulates the adaptive immune response in human limb ischaemia-reperfusion injury. *Br J Surg* 2009; **96**: 381-390.
67. Sablotzki A, Dehne M, Welters I, Menges T, Lehmann N, Gorlach G, et al. Alterations of the cytokine network in patients undergoing cardiopulmonary bypass. *Perfusion* 1997; **12**: 393-403.
68. Sablotzki A, Dehne MG, Mann V, Gorlach G, Muhling J, Zickmann B, et al. Plasma levels of selectins and interleukins in cardiovascular surgery using cardiopulmonary bypass. *Thoracic and Cardiovascular Surgery* 1999; **47**: 26-31.
69. Long H, Lin ZC, Situ DR, He LR, Yan SL, Lin YB, et al. [Cytokine responses after lobectomy: a prospective randomized comparison of video-assisted thoracoscopic surgery and minimal incision thoracotomy]. *Ai Zheng* 2007; **26**: 991-995.
70. Farsak B, Gunaydin S, Yildiz U, Sari T, Zorlutuna Y. Clinical evaluation of leukocyte filtration as an alternative anti-inflammatory strategy to aprotinin in high-risk patients undergoing coronary revascularization. *Surg Today* 2012; **42**: 334-341.
71. Murakami T, Iwagaki H, Saito S, Ohtani S, Kuroki K, Kuinose M, et al. Equivalence of the acute cytokine surge and myocardial injury after coronary artery bypass grafting with and without a novel extracorporeal circulation system. *J Int Med Res* 2005; **33**: 133-149.
72. Wang SY, Li TY, Zheng YQ. [Effects of cardiopulmonary bypass on plasma pentraxin-3 level and the balance of Th1/Th2 function in children with congenital heart malformations]. *Zhonghua Yi Xue Za Zhi* 2009; **89**: 1024-1027.
73. Servis D, Busic Z, Stipancic I, Patrlj L, Gagro A. Serum cytokine changes after gastric resection or gastrectomy for gastric cancer. *Hepatogastroenterology* 2008; **55**: 1868-1872.
74. Szerafin T, Niederpold T, Mangold A, Hoetzenecker K, Hacker S, Roth G, et al. Secretion of soluble ST2 - possible explanation for systemic immunosuppression after heart surgery. *Thoracic and Cardiovascular Surgery* 2009; **57**: 25-29.
75. Franke A, Lante W, Fackeldey V, Becker HP, Thode C, Kuhlmann WD, et al. Proinflammatory and antiinflammatory cytokines after cardiac operation: different cellular sources at different times. *Ann Thorac Surg* 2002; **74**: 363-370; discussion 370-361.
76. Fassbender K, Gerber B, Karrer U, Sobieska M, Aeschlimann A, Muller W. Glycosylation of acute phase proteins and interleukins following hip arthroplasty. Inflammation parameters studied in 10 patients. *Acta Orthop Scand* 1993; **64**: 216-220.
77. Sakai H, Mori K, Suzuki K, Katayama Y, Matsuyama T. [The clinical significance of interleukin-6 as an inflammatory marker (the studies in patients with open heart surgery)]. *Rinsho Byori* 1994; **42**: 1151-1157.
78. Kristiansson M, Soop M, Sundqvist KG, Soop A, Suontaka AM, Blomback M. Local vs. systemic immune and haemostatic response to hip arthroplasty. *Eur J Anaesthesiol* 1998; **15**: 260-270.

79. Speziale G, Ferroni P, Ruvolo G, Fattouch K, Pulcinelli FM, Lenti L, et al. Effect of normothermic versus hypothermic cardiopulmonary bypass on cytokine production and platelet function. *Journal of Cardiovascular Surgery (Torino)* 2000; **41**: 819-827.
80. Wirtz DC, Heller KD, Miltner O, Zilkens KW, Wolff JM. Interleukin-6: a potential inflammatory marker after total joint replacement. *Int Orthop* 2000; **24**: 194-196.
81. Fujioka S. Increased plasma concentration of adrenomedullin during and after major surgery. *Surg Today* 2001; **31**: 575-579.
82. Handel M, Winkler J, Hornlein RF, Northoff H, Heeg P, Teschner M, et al. Increased interleukin-6 in collected drainage blood after total knee arthroplasty: an association with febrile reactions during retransfusion. *Acta Orthop Scand* 2001; **72**: 270-272.
83. Wozniak W, Wierusz-Kozlowska M, Markuszewski J, Lesniewska K, Wiktorowicz K. [Monitoring of IL-6 levels in patients after total hip joint replacement]. *Chir Narzadow Ruchu Ortop Pol* 2004; **69**: 121-124.
84. Pasnik J, Moll JA, Moll J, Cywinska-Bernas A, Mazurowski W, Arendarczyk J, et al. [Alterations of procalcitonin and interleukin 6 after cardiopulmonary bypass in children with congenital heart disease]. *Polski Merkuriusz Lekarski* 2005; **19**: 20-23.
85. Takenaka K, Ogawa E, Wada H, Hirata T. Systemic inflammatory response syndrome and surgical stress in thoracic surgery. *J Crit Care* 2006; **21**: 48-53; discussion 53-45.
86. Moselli NM, Baricocchi E, Ribero D, Sottile A, Suita L, Debernardi F. Intraoperative epidural analgesia prevents the early proinflammatory response to surgical trauma. Results from a prospective randomized clinical trial of intraoperative epidural versus general analgesia. *Ann Surg Oncol* 2011; **18**: 2722-2731.
87. Honsawek S, Deepaisarnsakul B, Tanavalee A, Sakdinakiattikoon M, Ngarmukos S, Preativatanyou K, et al. Relationship of serum IL-6, C-reactive protein, erythrocyte sedimentation rate, and knee skin temperature after total knee arthroplasty: a prospective study. *Int Orthop* 2011; **35**: 31-35.
88. Chen N, Ma W, Shen WD. [Influence of different frequency electroacupuncture on serum IL-1 and IL-6 in patients with pneumonectomy]. *Zhongguo Zhen Jiu* 2012; **32**: 523-526.
89. Holzheimer RG, Molloy RG, Gorlach H, Wilkert S, Hehrlein F. IL-6 and TNF alpha release in association with neutrophil activation after cardiopulmonary bypass surgery. *Infection* 1994; **22**: 37-42.
90. Sakamoto K, Arakawa H, Mita S, Ishiko T, Ikei S, Egami H, et al. Elevation of circulating interleukin 6 after surgery: factors influencing the serum level. *Cytokine* 1994; **6**: 181-186.
91. Deng MC, Wiedner M, Erren M, Mollhoff T, Assmann G, Scheld HH. Arterial and venous cytokine response to cardiopulmonary bypass for low risk CABG and relation to hemodynamics. *European Journal Cardio-Thoracic Surgery* 1995; **9**: 22-29.
92. Kragsbjerg P, Holmberg H, Vikerfors T. Serum concentrations of interleukin-6, tumour necrosis factor-alpha, and C-reactive protein in patients undergoing major operations. *Eur J Surg* 1995; **161**: 17-22.
93. Kawahito K, Adachi H, Ino T. Influence of surgical procedures on interleukin-6 and monocyte chemotactic and activating factor responses: CABG vs. valvular surgery. *J Interferon Cytokine Res* 2000; **20**: 1-6.
94. Hogevold HE, Lyberg T, Kahler H, Haug E, Reikeras O. Changes in plasma IL-1beta, TNF-alpha and IL-6 after total hip replacement surgery in general or regional anaesthesia. *Cytokine* 2000; **12**: 1156-1159.

95. Takeda S, Nakanishi K, Ikezaki H, Kim C, Sakamoto A, Tanaka K, et al. Cardiac marker responses to coronary artery bypass graft surgery with cardiopulmonary bypass and aortic cross-clamping. *J Cardiothorac Vasc Anesth* 2002; **16**: 421-425.
96. Franke A, Lante W, Fackeldey V, Becker HP, Kurig E, Zoller LG, et al. Pro-inflammatory cytokines after different kinds of cardio-thoracic surgical procedures: is what we see what we know? *European Journal Cardio-Thoracic Surgery* 2005; **28**: 569-575.
97. de Mendonca-Filho HT, Pereira KC, Fontes M, Vieira DA, de Mendonca ML, Campos LA, et al. Circulating inflammatory mediators and organ dysfunction after cardiovascular surgery with cardiopulmonary bypass: a prospective observational study. *Critical Care* 2006; **10**: R46.
98. Tablov B, Stavreva S, Tablov V, Popov I, Konova E. [Immunological aspects of postoperative analgesia after abdominal hysterectomy]. *Akush Ginekol (Sofiia)* 2007; **46**: 18-24.
99. Torres A, Torres K, Paszkowski T, Staskiewicz GJ, Maciejewski R. Cytokine response in the postoperative period after surgical treatment of benign adnexal masses: comparison between laparoscopy and laparotomy. *Surg Endosc* 2007; **21**: 1841-1848.
100. Szczesny TJ, Slotwinski R, Stankiewicz A, Szczygiel B, Zaleska M, Kopacz M. Interleukin 6 and interleukin 1 receptor antagonist as early markers of complications after lung cancer surgery. *European Journal Cardio-Thoracic Surgery* 2007; **31**: 719-724.
101. Szczesny TJ, Slotwinski R, Szczygiel B, Stankiewicz A, Zaleska M, Kopacz M, et al. Systematic mediastinal lymphadenectomy does not increase postoperative immune response after major lung resections. *European Journal Cardio-Thoracic Surgery* 2007; **32**: 868-872.
102. Tamayo E, Alvarez FJ, Alonso O, Bustamante R, Castrodeza J, Soria S, et al. Effects of simvastatin on systemic inflammatory responses after cardiopulmonary bypass. *Journal of Cardiovascular Surgery (Torino)* 2009; **50**: 687-694.
103. Yoon SZ, Jang IJ, Choi YJ, Kang MH, Lim HJ, Lim YJ, et al. Association between tumor necrosis factor alpha 308G/A polymorphism and increased proinflammatory cytokine release after cardiac surgery with cardiopulmonary bypass in the Korean population. *J Cardiothorac Vasc Anesth* 2009; **23**: 646-650.
104. Sasajima K, Futami R, Matsutani T, Nomura T, Makino H, Maruyama H, et al. Increases in soluble tumor necrosis factor receptors coincide with increases in interleukin-6 and proteinases after major surgery. *Hepatogastroenterology* 2009; **56**: 1377-1381.
105. Di Vita G, Patti R, Fama F, Balistreri CR, Candore G, Caruso C. Changes of inflammatory mediators in obese patients after laparoscopic cholecystectomy. *World J Surg* 2010; **34**: 2045-2050.
106. Sun YJ, Song DD, Diao YG, Zhou J, Zhang TZ. Penehyclidine hydrochloride preserves the intestinal barrier function in patients undergoing cardiopulmonary bypass. *J Thorac Cardiovasc Surg* 2013; **146**: 179-185.
107. Narita S, Tsuchiya N, Kumazawa T, Maita S, Numakura K, Obara T, et al. Comparison of surgical stress in patients undergoing open versus laparoscopic radical prostatectomy by measuring perioperative serum cytokine levels. *Journal of Laparoendoscopic and Advanced Surgical Techniques Part A* 2013; **23**: 33-37.
108. Liu DQ, Lu Y, Wang MY. [Research on the expression of some immune markers after reamed and unreamed femoral nailing]. *Zhonghua Wai Ke Za Zhi* 2004; **42**: 741-745.
109. Kobayashi M, Tsujitani S, Kurisu Y, Kaibara N. Responses of cytokines and coagulation-fibrinolytic states to surgical stress following esophagectomy. *Hepatogastroenterology* 2004; **51**: 1376-1378.

110. Nakamura K, Ueno T, Yamamoto H, Iguro Y, Yamada K, Sakata R. Relationship between cerebral injury and inflammatory responses in patients undergoing cardiac surgery with cardiopulmonary bypass. *Cytokine* 2005; **29**: 95-104.
111. Scheingraber S, Dobbert D, Schmiedel P, Seliger E, Dralle H. Gender-specific differences in sex hormones and cytokines in patients undergoing major abdominal surgery. *Surg Today* 2005; **35**: 846-854.
112. Kremen J, Dolinkova M, Krajickova J, Blaha J, Anderlova K, Lacinova Z, et al. Increased subcutaneous and epicardial adipose tissue production of proinflammatory cytokines in cardiac surgery patients: possible role in postoperative insulin resistance. *J Clin Endocrinol Metab* 2006; **91**: 4620-4627.
113. Tamayo E, Alvarez FJ, Alonso O, Castrodeza J, Bustamante R, Gomez-Herreras JI, et al. The inflammatory response to colloids and crystalloids used for pump priming during cardiopulmonary bypass. *Acta Anaesthesiol Scand* 2008; **52**: 1204-1212.
114. Cesari F, Caporale R, Marcucci R, Caciolli S, Stefano PL, Capalbo A, et al. NT-proBNP and the anti-inflammatory cytokines are correlated with endothelial progenitor cells' response to cardiac surgery. *Atherosclerosis* 2008; **199**: 138-146.
115. Zingg U, Forberger J, Frey DM, Esterman AJ, Oertli D, Beck-Schimmer B, et al. Inflammatory response in ventilated left and collapsed right lungs, serum and pleural fluid, in transthoracic esophagectomy for cancer. *Eur Cytokine Netw* 2010; **21**: 50-57.
116. Hsing CH, Hsieh MY, Chen WY, Cheung So E, Cheng BC, Chang MS. Induction of interleukin-19 and interleukin-22 after cardiac surgery with cardiopulmonary bypass. *Ann Thorac Surg* 2006; **81**: 2196-2201.
117. Kim KT, Lee SH, Suk KS, Bae SC. The quantitative analysis of tissue injury markers after mini-open lumbar fusion. *Spine (Phila Pa 1976)* 2006; **31**: 712-716.
118. Dimopoulou I, Armaganidis A, Douka E, Mavrou I, Augustatou C, Kopterides P, et al. Tumour necrosis factor-alpha (TNFalpha) and interleukin-10 are crucial mediators in post-operative systemic inflammatory response and determine the occurrence of complications after major abdominal surgery. *Cytokine* 2007; **37**: 55-61.
119. Kotulak T, Drapalova J, Kopecky P, Lacinova Z, Kramar P, Riha H, et al. Increased circulating and epicardial adipose tissue mRNA expression of fibroblast growth factor-21 after cardiac surgery: possible role in postoperative inflammatory response and insulin resistance. *Physiol Res* 2011; **60**: 757-767.
120. Kim JC, Shim JK, Lee S, Yoo YC, Yang SY, Kwak YL. Effect of combined remote ischemic preconditioning and postconditioning on pulmonary function in valvular heart surgery. *Chest* 2012; **142**: 467-475.
121. Svitek V, Lonsky V, Mandak J, Krejsek J, Kolackova M, Brzek V, et al. No clear clinical benefit of using mini-invasive extracorporeal circulation in coronary artery bypass grafting in low-risk patients. *Perfusion* 2009; **24**: 389-395.
122. Gormley SM, McBride WT, Armstrong MA, Young IS, McClean E, MacGowan SW, et al. Plasma and urinary cytokine homeostasis and renal dysfunction during cardiac surgery. *Anesthesiology* 2000; **93**: 1210-1216; discussion 1215A.
123. Gormley SM, McBride WT, Armstrong MA, McClean E, MacGowan SW, Campalani G, et al. Plasma and urinary cytokine homeostasis and renal function during cardiac surgery without cardiopulmonary bypass. *Cytokine* 2002; **17**: 61-65.
124. Alatas O, Colak O, Buyukkidan B, Portakal O, Tanriverdi B. Soluble interleukin-2 receptor and interleukin-8 plasma levels during and after cardiopulmonary bypass: correlations with creatine kinase and creatine kinase MB. *Clin Exp Med* 2001; **1**: 13-18.

125. Krohn CD, Reikeras O, Aasen AO. The cytokines IL-1beta and IL-1 receptor antagonist, IL-2 and IL-2 soluble receptor-alpha, IL-6 and IL-6 soluble receptor, TNF-alpha and TNF soluble receptor I, and IL10 in drained and systemic blood after major orthopaedic surgery. *Eur J Surg* 1999; **165**: 101-109.

126. Jedynak M, Mroczko B, Siemiatkowski A, Gacko M, Szmitkowski M. Decreased serum level of IL-12 in the course of ischemia and reperfusion during abdominal aortic surgery. *Folia Histochem Cytobiol* 2011; **49**: 465-471.

127. Szerafin T, Brunner M, Horvath A, Szentgyorgyi L, Moser B, Boltz-Nitulescu G, et al. Soluble ST2 protein in cardiac surgery: a possible negative feedback loop to prevent uncontrolled inflammatory reactions. *Clinical Laboratory* 2005; **51**: 657-663.

128. Kudlova M, Kolackova M, Kunes P, Andrys C, Jankovicova K, Mand'ak J, et al. [Cardiac surgery operations and their influence on serum level of antiinflammatory cytokine interleukin-10]. *Cas Lek Cesk* 2007; **146**: 48-55; discussion 55-46.

129. Jiang S, Liu X, Luo L, Qu B, Huang X, Lin Y, et al. Serum levels of Th17-related cytokines in Behcet disease patients after cataract surgery. *Mol Vis* 2011; **17**: 1425-1430.

130. Thiele RI, Daniel V, Opelz G, Lange R, Sack FU, Jakob H, et al. Circulating interleukin-1 receptor antagonist (IL-1RA) serum levels in patients undergoing orthotopic heart transplantation. *Transpl Int* 1998; **11**: 443-448.

131. Ye DX, Xu Q, Chen WT, Zhu HG, Lin GC, Jiang CH, et al. [Immunodetections and analysis of peripheral blood for patients with oral cancer undergoing operation]. *Shanghai Kou Qiang Yi Xue* 2004; **13**: 83-86.

132. Wehlin L, Vedin J, Vaage J, Lundahl J. Peripheral blood monocyte activation during coronary artery bypass grafting with or without cardiopulmonary bypass. *Scand Cardiovasc J* 2005; **39**: 78-86.

133. Kudlova M, Kunes P, Kolackova M, Lonsky V, Mandak J, Andrys C, et al. Lipopolysaccharide binding protein and sCD14 are not produced as acute phase proteins in cardiac surgery. *Mediators Inflamm* 2007; **2007**: 72356.

134. Suda K, Kitagawa Y, Ozawa S, Saikawa Y, Ueda M, Abraham E, et al. Serum concentrations of high-mobility group box chromosomal protein 1 before and after exposure to the surgical stress of thoracic esophagectomy: a predictor of clinical course after surgery? *Diseases of Esophagus* 2006; **19**: 5-9.

135. Giannoudis PV, Mallina R, Harwood P, Perry S, Sante ED, Pape HC. Pattern of release and relationship between HMGB-1 and IL-6 following blunt trauma. *Injury* 2010; **41**: 1323-1327.

136. Takahata R, Ono S, Tsujimoto H, Hiraki S, Kimura A, Kinoshita M, et al. Postoperative serum concentrations of high mobility group box chromosomal protein-1 correlates to the duration of SIRS and pulmonary dysfunction following gastrointestinal surgery. *J Surg Res* 2011; **170**: e135-140.

137. Osoegawa A, Yano T, Yamanaka T, Tagawa T, Shoji F, Yoshino I, et al. Plasma high-mobility group box 1 as an indicator of surgical stress. *Surg Today* 2011; **41**: 903-907.

138. Kohno T, Anzai T, Shimizu H, Kaneko H, Sugano Y, Yamada S, et al. Impact of serum high-mobility group box 1 protein elevation on oxygenation impairment after thoracic aortic aneurysm repair. *Heart Vessels* 2011; **26**: 306-312.

139. Szerafin T, Hoetzenecker K, Hacker S, Horvath A, Pollreisz A, Arpad P, et al. Heat shock proteins 27, 60, 70, 90alpha, and 20S proteasome in on-pump versus off-pump coronary artery bypass graft patients. *Ann Thorac Surg* 2008; **85**: 80-87.

140. Pizon MT, Gburek T, Pizon M, Sztefko K. Kinetics of plasma heat shock protein HSP-70 release in coronary artery surgery: on-pump versus off-pump. *Minerva Chir* 2006; **61**: 483-491.
141. Dybdahl B, Wahba A, Lien E, Flo TH, Waage A, Qureshi N, et al. Inflammatory Response After Open Heart Surgery: Release of Heat-Shock Protein 70 and Signaling Through Toll-Like Receptor-4. *Circulation* 2002; **105**: 685-690.
